# Evolving Always-Critical Networks

**DOI:** 10.3390/life10030022

**Published:** 2020-03-04

**Authors:** Marco Villani, Salvatore Magrì, Andrea Roli, Roberto Serra

**Affiliations:** 1 Department of Physics, Informatics and Mathematics, University of Modena and Reggio Emilia, I-41125 Modena, Italy; rserra@unimore.it; 2 European Centre for Living Technology, 30123 Venice, Italy; andrea.roli@unibo.it; 3 Department of Physics, University of Bologna, 40126 Bologna, Italy; salvamagri95@gmail.com; 4 Department of Computer Science and Engineering, University of Bologna, 40126 Bologna, Italy; 5 Institute for Advanced Study, University of Amsterdam, 1012 WX Amsterdam, The Netherlands

**Keywords:** evolving systems, criticality, edge of chaos, gene regulatory networks, Boolean models, genetic algorithms, random Boolean networks

## Abstract

Living beings share several common features at the molecular level, but there are very few large-scale “operating principles” which hold for all (or almost all) organisms. However, biology is subject to a deluge of data, and as such, general concepts such as this would be extremely valuable. One interesting candidate is the “criticality” principle, which claims that biological evolution favors those dynamical regimes that are intermediaries between ordered and disordered states (i.e., “at the edge of chaos”). The reasons why this should be the case and experimental evidence are briefly discussed, observing that gene regulatory networks are indeed often found on, or close to, the critical boundaries. Therefore, assuming that criticality provides an edge, it is important to ascertain whether systems that are critical can further evolve while remaining critical. In order to explore the possibility of achieving such “always-critical” evolution, we resort to simulated evolution, by suitably modifying a genetic algorithm in such a way that the newly-generated individuals are constrained to be critical. It is then shown that these modified genetic algorithms can actually develop critical gene regulatory networks with two interesting (and quite different) features of biological significance, involving, in one case, the average gene activation values and, in the other case, the response to perturbations. These two cases suggest that it is often possible to evolve networks with interesting properties without losing the advantages of criticality. The evolved networks also show some interesting features which are discussed.

## 1. Introduction

Nature displays “endless forms most beautiful” [1], but the diversity is much lower when observed at the molecular level, where all living systems share many common features, including inter alia: they are all made of cells; they are mostly composed of lipids, proteins, and nucleic acids; proteins are synthesized through the well-known DNA–RNA-ribosome process; catalysis is performed by enzymes; DNA stores genetic information, moreover a *universal* code is used. These are just some examples, and while there are other features that are not completely universal, most are nonetheless shared by large classes of species (for example, the use of ATP as an energy carrier).

While such universal microscopic features have been well-known for decades, there are very few large-scale “operating principles” which seem to hold for all living systems. The most prominent such principle is, of course, biological evolution; unfortunately there are not many general principles of this kind. Such principles would be welcome, in an era that is generating an unprecedented deluge of biological data. The great times in science are those where data and theories grow hand-in-hand, as happened with quantum mechanics in the first half of the 20^th^ century, or to molecular biology in the years which followed the end of the Second World War. On the other hand, progress is slow when there is an imbalance between the richness of data and that of theories. Nowadays, in fundamental physics, there are various elegant and sophisticated theories that seem far from possible experimental verification in the foreseeable future, while in biology, the reverse is true, since there is an overabundance of data, with very few general principles by which to interpret them. Therefore, such general concepts would be extremely valuable, and we will focus on one interesting candidate, which will be called the “criticality” principle (CP) in this paper.

This principle is based on the idea that living beings are dynamical systems, and that there are some dynamical regimes that are favored by biological evolution [2,3]. It is well-known that, depending upon the values of some parameters, the behavior in time of dynamical systems can either be ordered (as in the case of a constant state or of a repeating cycle of states) or disordered (as in the case of chaotic states, which look like noisy signals and display sudden jumps between widely varying values). There are regions in parameter space that correspond to order, and regions that correspond to disorder. The surfaces that separate these different regimes are the critical regions, and the states of these surfaces are critical.

Therefore, critical states are in-between ordered and disordered ones. A suitable way of describing them is that they are “at the edge of chaos” [3,4,5,6,7]. The criticality principle states that these states are at an advantage with respect either to chaotic states, since they are more stable and controllable, or to ordered states, since they can better change in response to different conditions, without being deeply stuck in the same state. If this is indeed the case, evolution should have modified the parameters in such a way that living beings are found near critical states—a statement that is, in principle, amenable to experimental verification.

An in-depth discussion of the criticality principle is beyond the scope of the present paper, and the interested reader is referred to the large body of scientific literature; see e.g., [8,9] for a review and for further bibliography. In this paper, we will limit ourselves to considering the issue of criticality in gene regulatory networks (GRNs), although the criticality principle applies to living systems as wholes, and the criticality of the whole organism does not imply that of all its subsystems. However, it is interesting to explore criticality in GRNs, with the caveat that the previous observation might imply some distance from criticality in this particular subsystem (although we might tentatively expect that these differences, if any, are of limited size).

In Section 2, we will quickly review a widely-used model of gene regulation, based upon the simplifying assumption that genes can be either on or off, referring the reader also in this case to the extensive scientific literature on Boolean models of GRNs [2,3,10,11]. By modelling some biological phenomena or systems with Boolean networks, it is possible to infer information about their dynamical regimes, and we will review data which, although not conclusive, suggest that biological organisms tend indeed to be found in, or pretty close to, critical states, thus supporting the validity of the CP [12,13,14,15,16].

The work described in this paper is mostly concerned with understanding whether critical systems can evolve while remaining critical. A species that is in a critical state will change in time, due to evolutionary pressure, and if the criticality principle really holds, it should continue to be critical in the various stages of its evolution; otherwise, it might become less effective and be at a selective disadvantage. We will refer to the above condition as “always-critical” evolution.

In order to explore the possibility of achieving always-critical evolution, one must resort to simulated evolution. Indeed, it must be observed that the criticality principle is relevant not only for biological systems, but also for artificial evolving systems, like, e.g., those studied in evolutionary computation [17,18]. Note that it is possible to simulate the behavior of systems which can change in time not only the value of some parameters, but also their own structure (e.g., by adding or removing variables, or interaction terms). Moreover, simulating the evolution of artificial systems can help us to better understand the power and limitations of the principle.

In Section 3, we consider the evolution of Boolean models of gene regulatory networks (Boolean networks (BNs)), like those of Section 2, which are required to reach a state with some peculiar features or to perform a given task. The approach is quite straightforward: a fitness function is introduced to measure the performance of the network, and a metaheuristics (almost always a genetic algorithm) is used to evolve the (population of) network(s). Starting from purely random BNs (RBNs), the evolution leads to the emergence of networks that are no longer completely random. Some previous works, also reviewed in Section 3, demonstrate that the developed artificial networks are not necessarily critical, and the criticality principle does not always hold in these cases.

This should not be surprising, given that critical systems are supposed to be superior to others in a changing environment, where it is important to be able to react to external modifications. If the task is constant, an ordered network might well outperform a critical one. Moreover, there are cases where some features of the adaptation process tend to modify the dynamical regime of the evolved networks as a side effect, e.g., as it will be shown in Section 4, by changing the ratio of “1s” in the truth tables, thus affecting the dynamical regime.

So, there is no reason to believe that an artificial critical system will perform better than an ordered or chaotic one, unless the features of the task actually favor criticality. Let us recall that, however, several observations serve as the bases of the CP, which show that biological systems or subsystems tend to be critical. This might tentatively be explained by the higher evolvability of critical systems, which would be hard to prove in biology, but which can be proposed on the basis of experiments on artificial systems. Let us mention here the work of [19], who studied the evolvability of RBNs, evaluating their ability to introduce innovations (i.e., new attractors) without destroying previous capabilities (i.e., by keeping most of the old attractors). The results of numerical experiments show that critical networks can achieve these goals better than others.

This leads us back to the central theme of this paper, i.e., the evolvability of critical networks, which are influenced by several important factors that can lead the system away from criticality. Let us suppose that a species is dynamically critical, but is subject to long-term modifications which alter the fitness of various individuals (e.g., due to changes of its environment). As discussed above, it is interesting to understand whether it is possible to adapt to these changes while remaining critical in all the steps of the evolutionary process, i.e., to explore the possibility of obtaining “always-critical” gene regulatory networks. It would therefore be useful to modify the evolutionary algorithms by constraining newly-generated networks to stay on a critical boundary (i.e., a set of states whose points are all critical). In Section 4.1, we discuss a modification of standard genetic algorithms (briefly, GAs) [20] that was devised so that the new individuals that are generated are critical, or close to criticality. As it will be shown, this can indeed be done, but leaves open the question of whether this further constraint will impair the system’s ability to perform well.

In order to explore this issue, in Section 4.2, we introduce two nontrivial tasks with interesting biological significance, i.e., the development of networks i) where the number of active genes is a predetermined fraction of the total, and ii) where the number of nodes in an “avalanche” which are “up” (i.e., whose activation values are larger than those of the wild type) is a given fraction of the total. An “avalanche” of gene perturbations is precisely defined in the text (Section 2), where it is also shown that the size distribution of avalanches depends upon the system dynamical state (ordered, critical or disordered). Task (i) is motivated by the fact that in multicellular organisms, in different cell types, the number of active genes is much smaller than the total number of genes, while task (ii) is motivated by the experimental observation, in the yeast S. Cerevisiae, of an abundance of “up-regulated” genes. Both features are not captured by fully random Boolean networks.

In Section 5, we use the modified GA of Section 4.1 to show that, starting from RBNs, populations of BNs can evolve to solve the two tasks of Section 4.2 while remaining critical. These results are very promising and were unexpected; they show that the evolution of always-critical networks is indeed possible in two different tasks which are both biologically relevant.

The networks at the end of their evolution are still critical, but they are no longer fully random, since they are able to perform the required task. These networks should therefore have some kind of (dynamical) structure that allows them to do so. However, as discussed in Section 5 and, in more detail, in Appendix A, it has not been possible so far to identify, in all cases, some relatively simple features that characterize the populations of evolved networks, so it is not yet possible to make suggestions about the possible general structural properties of successful evolved networks.

Section 6 is dedicated to a critical revision of the results obtained, to some comments about the method that has been used, and to a summary of future work.

## 2. Boolean Models of Gene Regulatory Networks

The regulation of gene expression is of the utmost importance, either in single cells or in multicellular organisms. In the former case, it allows the cell to adapt to various conditions, like, e.g., the availability of nutrients, while in the latter case, it is responsible for the existence of different cell types sharing the same genome.

An intriguing feature of this process is that the expression of a gene regulates, and is regulated by, the expression of other genes, through various complex effects, including in particular the synthesis of proteins (or of other gene products, like small RNAs) which can bind to the DNA and promote or inhibit the expression of some genes. Moreover, the presence of some enzymes affects the presence in the cell of small chemicals, which, in turn, may bind—or not—to some protein, thus modifying its regulatory properties.

While there are detailed models of the various steps of the whole process, we are interested here in the overall outcomes of all these interactions, which are better captured by strongly simplified models that describe the effects of the process of regulation from a more remote perspective, rejecting the idea of describing every detail.

The better known models of this type are those of gene regulatory networks, which describe a network of interacting genes only, without explicitly taking into account the other entities like proteins, small RNAs, etc., whose influence is subsumed by the interaction rules among the genes. In order to simplify the writing (and the modelling!), we will assume that each node is a “gene” and each product is a “protein”, as in textbook examples, although the nodes of a biological network may be portions of the genome with different properties, which can give rise to different kinds of products (e.g., miRNA).

There is an activation value associated to each “gene”, which may describe the density (or copy number) of the corresponding “protein”. There are models where activations can take continuous values (or sometimes a set of discrete values), and we know from various measuring methods that the expression levels can indeed be very different. However, many interesting results have been obtained by adopting a Boolean description, which we will do here. This choice naturally leads to a discrete-time model, rather than a continuous one [2,3].

So, we have narrowed our interest to time-discrete Boolean models which are able to deal with the activation patterns of several genes. We also need to distinguish two different kinds of models of this type:

- models of specific genetic circuits in specific organisms, including, e.g., the mammalian cell cycle, the T-cell differentiation, the yeast apoptosis, and others. We will not refer to individual papers here, but to the Cell Collective repository [21] where several models of this kind are available, including, e.g., the mammalian cell cycle, the T-cell differentiation, yeast apoptosis, the Arabidopsis thaliana cell cycle, and many others. These networks are formed on the basis of detailed biological information, which can come from static or dynamic knowledge. One limitation is that our present knowledge of any genetic circuit is limited, so the model might miss important links, or create spurious ones. Moreover, the specific networks comprise a number of variables which ranges from about ten to a few hundred variables, while the overall gene network of the organism is composed of thousands or tens of thousands of variables. In order to take this into account, models of this kind include genes which represent the effects of the rest of the network (and of the external environment), i.e., nodes whose values are given from outside the model (they are not regulated by what happens in the network)
- “generic” models, which do not try to describe any particular organism, but rather, aim at understanding general properties, common to different organisms [2,3,12,22,23]. The questions that can be addressed by these models are mostly related to how different properties scale with the size of the network, or with its topology, the type of gene–gene relationships that are possible, etc. In these models, randomness plays a key role. There may be some constraints based upon the available biological knowledge, but the main way to uncover generic properties is to allow a number of model choices to vary at random, and to collect the results of a large ensemble of cases.

Let us note that random does not necessarily mean “with uniform probability”; this depends upon the model and the known constraints. But, for the sake of definiteness, let us focus on a peculiar class of generic models, i.e., random Boolean networks (RBNs for short) which started this line of research about 50 years ago. In this case, the network topology is really random: the number of connections per node is fixed, but which nodes are connected is determined by chance, with uniform probability. The Boolean functions associated to each node are also drawn at random.

RBNs have a long history, and there are several slightly different models of this kind. For the sake of definiteness, we will refer to “classic” RBNs [2,10,11,24] as described below.

Formally, BNs are discrete-time and discrete-state dynamical systems. Following their original formulation, they can be represented by a directed graph with ***N*** nodes, each having an associated Boolean variable, *x_i_*, *i* = 1,...,***N***, and a Boolean function *f_i_* (*x_i1_*,...,*x_ik_*) which depends on *k* other nodes.

Let ***X*** = (*x_1_*...*x_N_*) denote the global state of the network. The value at time *t* + 1 of the *i*-th node, i.e., *x_i_*(*t*+1), is determined by the value of *f_i_*(*x_i1_*,...,*x_ik_*) computed at the previous time step, i.e., *x_i_*(t+1)=*f_i_*(*x_i1_*(*t*),...,*x_ik_*(*t*)

Random BNs (RBNs) comprises the most prominent class, in which functions and connections are chosen according to predefined distributions. In the case considered in this paper:

- the topology and the Boolean functions are fixed in time
- each node receives exactly *k* distinct inputs from *k* other nodes chosen at random with uniform probability among the remaining *n −* 1 nodes (avoiding self-loops and multiple connections)
- the Boolean functions are defined by choosing at random, for each of the 2*^k^* entries of the truth table, the value 1 with probability *p* (called the bias). The Boolean function associated with a node is chosen independently from those of other nodes
- the updating is synchronous, so ***X*** (*t* + 1) is a deterministic function of ***X*** (*t*)

Finite RBNs are finite-state deterministic systems; therefore, they always end up in cyclic states (a fixed point being a particular case of a cycle whose period has length 1). Extensive simulations and some limited analytical results have shown that RBNs exhibit a kind of phase transition between order and chaos depending on the values of *k* and p. For 2*p* (1 *− p*) *k* < 1, RBNs have, on average, an “ordered” behavior, i.e., the cycles have quite regular basins of attraction, whose length grows with the number of nodes in a polynomial way. On the other hand, for 2*p* (1 *− p*) *k* > 1, the networks show extreme sensitivity to the initial conditions and very long cyclic attractors, so they are called “chaotic” (although the asymptotic states are actually cycles). A critical regime is attained for 2*p* (1 *− p*) *k* = 1.

RBNs are particularly interesting as models of complex systems, since their dynamical behavior can be tuned by modifying connections and Boolean functions.

Interestingly, these different behaviors can be related to the value of a single index, often called the Derrida parameter *λ* [3,25,26]. On average, in “ordered” networks (*λ* < 1), a small perturbation tends to vanish, while in chaotic networks (*λ* > 1), it increases in time, and in critical networks (*λ* = 1), it tends to maintain its size. The Derrida parameter is related to the discrete analogue of the Lyapunov exponent that is often used to analyze the stability of continuous dynamical systems [27].

The Derrida parameter can be determined by studying the average behavior in time of the distance between two close initial states of a network, and taking its limiting value when the initial distance approaches its minimum value (dynamical sensitivity). In practice, one often considers the short time evolution of the distance between two initial states that differ by a single spin flip. On the other hand, it is also possible to estimate the value of the sensitivity in a static way without performing dynamical simulations [28]. This can be achieved by considering the downstream effect of a single flip on the input of the various Boolean functions. The choice of the most representative value of the sensitivity may be complicated [29] but, in the case of ergodic systems, the dynamic and static methods asymptotically provide the same estimate (while the effective values computed on finite numbers of cases may of course differ).

As discussed in Section 1, several authors have suggested that GRNs should be found in, or close to, critical states. In principle, the dynamical criticality of a GRN might be explored by slightly perturbing the activation of one or a few genes, and looking at the time behavior of the network of N genes. However, direct observation of this phenomenon, with the relevant time resolution, still seems far from sight. Luckily, using plausible assumptions, it is often possible to circumvent this problem. In the case of several models of type (a), which describe specific genetic circuits, a wide analysis by [15] has shown that the (static) sensitivity is close to 1. Similar results were obtained using models of type (b), i.e., RBNs, which were applied to describe the effects of gene perturbations induced by permanently inhibiting the expression of a single gene (knock-out) and by observing the changes of the expression levels of the other genes [12,14,16,30].

For a given knock-out, let us define a gene as “affected” if its value is significantly modified with respect to the wild type. Since we use a Boolean description, a gene is either affected or not. This requires, of course, a suitable way to binarize the continuous values. Let then an avalanche be the set of affected genes, and define the size of the avalanche as the fraction of all the genes that are affected. The process of knock-out can be simulated in RBNs [30], and it is possible to demonstrate that, under suitable assumptions, the size distribution depends only upon the Derrida parameter. In this way, it is possible to use a static measure (the size of avalanches) to draw inferences about the dynamical regime of the network. This has been discussed in a series of papers, and it has been shown that it works in the well-studied case of the yeast S. Cerevisiae [12,14,16]. The best estimate of the Derrida parameter places it in the ordered region, quite close to the critical boundary [16].

In synthesis, these studies suggest that biological GRNs may indeed be critical, or close to criticality, thus providing support (although the evidence is not conclusive) to the CP.

## 3. Evolving Boolean Networks

The complex and rich dynamics that characterize BNs are likely to be one of the reasons for their suitability in modelling biological cells. This complexity can be further exploited when BNs are subject to an evolutionary process that, akin to natural selection, iteratively manipulates populations of BNs to produce networks with high fitness, i.e., high values of a given merit factor. The general scheme of such a process is the following: some individuals of the current population are selected (i.e., sampled with a bias in favor of individuals with higher fitness), and they are used as blueprints to generate an offspring of new individuals by recombination, which are, in turn, subject to mutation so as to introduce random variations of the original genetic material; then, a new population is composed, often by keeping some of the best individuals between parent population and offspring. Many instances of such a general scheme can be devised and the applications of evolutionary algorithms are countless [31].

To the best of our knowledge, the first work explicitly dealing with the artificial evolution of BNs is by Kauffman and Smith [32]. In that work, the goal is to evolve BNs such that their attractors match a prescribed target state. The algorithm used is analogous to a stochastic ascent local search, which can be considered as a minimalistic evolutionary algorithm: a single individual is iteratively modified by mutation (either a connection is randomly rewired or a bit in a function truth table is flipped), and only mutations producing a fitter individual are kept. RBNs with *k* = 2 and *k* = 10 are used in the experiments. An interesting observation made in the paper is that in networks with *k* = 2, the neighbors of an individual that are obtained by applying a mutation to the connections have similar fitness values, whilst in networks with *k* = 10, the fitness of neighbors is almost indistinguishable from that of random samples. This result can be explained in terms of *NK* models, in which the correlation of the fitness landscape decreases with *k* [2]. Lemke et al. [33] address a similar problem (i.e., the networks are required to match a target attractor) applying a genetic algorithm with mutations on both Boolean functions and connections, and crossover. They experiment with RBNs with *k* in {1,2,...,6}. Contrary to the results presented in [32], Lemke and co-authors do not observe in networks with *k* = 2 a qualitative behavior different from *k* > 2, and conclude that they have not found experimental evidence to support the conjecture that “adaptation on the edge of chaos is facilitated”.

A genetic algorithm with both crossover and mutation is used in [34] to obtain BNs with prescribed attractor lengths. The genetic operators modify only the Boolean functions of BNs with *k =* 3, while the topology is randomly set and remains unchanged. An interesting outcome of the work comes from the comparison between the initial and final average Boolean function bias along the evolutionary process. According to the formula *2p* (1 *− p*)*k* = 1 mentioned in Section 2, the initial average bias turns out to be approximately equal to 0.85 for ordered networks, 0.79 for critical, and 0.5 for chaotic ones; the results show that the bias tends to decrease in ordered and critical BNs, while it remains at 0.5 for chaotic ones. This phenomenon is common to all the works we have previously surveyed, and it can be explained by considering that the impact of random mutation on Boolean functions is to push the distribution of 0 s and 1 s towards an equal fraction of the two values. We note that the question as to whether the final BNs still belong to the initial dynamical regime is, of course, another matter, as, after evolution, the distribution of the Boolean functions can be different from the uniform one. As a consequence, whilst the works discussed so far more or less explicitly mention the CP, they merely elaborate on the relation between *k* and *p*, which provides a prediction of the dynamical regime that holds only for random BNs; therefore, the relationship between the performance of the evolutionary process and dynamical regime cannot be assessed. A work that explicitly takes into account the dynamical regime of BNs is that of Esmaeili and Jacob [35], who evolve populations of RBNs aiming to attain networks which are robust to perturbations. To achieve this objective, the genetic algorithm they devise tries to maximize a fitness function defined by the sum of several merit factors measuring features such as network sensitivity, number of attractors and basin entropy. (The information processing capacity of a complex dynamical system is reflected in the partitioning of its state space into disjoint basins of attraction, with state trajectories in each basin flowing towards its corresponding attractor. The basin entropy is a measure of the information that such a system is capable of storing, based on the length of the attractors and the number of basin states draining into that attractor; see [36] for details.) The maximization of a fitness function that contains a basin entropy component is expected to push the network towards the edge of chaos. The authors found that the most robust networks are characterized by a tendency towards order. This result is not surprising, because the fitness function is defined with the main goal of attaining robustness, which can easily be achieved in ordered networks, while the component related to basin entropy has the effect of keeping networks close to criticality. In conclusion, even if the dynamical regime of BNs is properly taken into account by considering a metric related to the dynamical properties of the network and not just static ones such as *k* and *p*, the goal of the work was not to challenge the CP.

The possibility of evolving robust BNs was thoroughly investigated in [37,38,39,40]. In those works, different variants of artificial evolution are used, from simple adaptive walks to genetic algorithms with both mutation and crossover, and variants of BNs are considered. In particular, in [41], generalized Derrida plots are introduced to investigate the dynamical regime of evolved networks. The original version of Derrida plots shows the average trend of a perturbation of size *h* (with 1 ≤ *h* ≤ *N −* 1) on a randomly chosen initial state of a BN after one updating step of the network. More precisely, the plots shows *h* (1) as a function of *h* (0). Generalized Derrida plots are defined for *t* ≥ 1 updating steps, i.e., they show *h* (*t*) with respect to *h* (0). BNs are evolved to be robust against perturbations on node values when the BN is on an attractor. The most notable result achieved is that the evolved BNs show a very different behavior with respect to random ones, and show a mixture of features which is typical of all the dynamical regimes. First of all, they are characterized by a small number of attractors, dominated by one short cyclic attractor with a large basin of attraction, which is typical of ordered RBNs. In addition, they show an initial slope in the Derrida plot similar to chaotic RBNs whilst, notably, the Derrida plot computed by starting from states belonging to an attractor is similar to that of critical RBNs.

It is worth noting that in the works we mentioned, evolutionary techniques are used as tools for producing one (or more) BN that satisfies requirements that depend upon the goal at hand. In this respect, artificial evolution is akin to the component that modifies the system’s parameters in a learning process; therefore, any technique that can be used to automatically design a target BN and applied and any metaheuristic technique [42,43] can be employed. The dynamical properties of evolved BNs are explored by applying both stochastic local search and genetic algorithms in [43], where several objectives are tested in the automatic design of BNs, from BNs whose trajectories satisfy given requirements (e.g., meeting a state in a given number of steps), to the density classification problem, in which the BN acts as a classifier that must be able to divide the initial states in two classes, depending on the majority of 0 s or 1 s. One of the contributions of that work is to stress the fact that as long as static tasks are considered and the fitness function does not depend upon specific dynamical BN conditions, one should not expect that critical BNs perform better than ordered and chaotic ones. Indeed, a good predictor for performance is simply the auto-correlation of the fitness landscape, which, being the result of fitness function, mutation operators or, in general, moves (i.e., limited perturbations to a solution that lead to neighboring solutions), is not necessarily related to the dynamical BN condition.

The automatic design of BNs matching some target properties of biological cells, such as the distance between attractors or the capability of producing attractor landscapes with specific characteristics resembling differentiation trees, is the subject of more recent works [44,45,46]. Finally, it is worth mentioning that evolutionary algorithms and stochastic local search techniques in general have been also applied to design BNs which are capable of controlling robots [47,48], and the structural and dynamical properties of these BNs have been studied as well [49,50,51].

We recognize that none of the aforementioned works explicitly constrains the dynamical regime of the networks along the evolutionary process. To the best of our knowledge, the first works applying this constraint are [52,53], where genetic algorithm variants with genetic operators that do not change the proportion of 0 s and 1 s in the Boolean functions are presented. Some of the results of these papers will be summarized below, as they are the starting point of this contribution.

We conclude this brief summary of related work by mentioning some more recent works: Taou et al. present a study in which BNs are evolved to control trajectories in other BNs [54]. The relationship between modularity and evolvability is investigated in [55] by evolving populations of threshold networks, and in [56], where the importance of rewiring mutations is emphasized.

## 4. Genetic Algorithms and the Case Studies

### 4.1. The Genetic Algorithms

In order to induce an evolutionary process, we make use of the well-known Genetic Algorithms (GAs) introduced by John Holland [20] (here, we assume that the reader is familiar with these techniques). The individuals are single BNs, the genotype being composed by the sequence of all Boolean tables that guide the dynamic response of the BN’s nodes; therefore, in each GA series, all BNs share the same topology; since the position of nodes is arbitrary, and the fact that we perform several GA series, this does not limit the set of different networks that can be generated.

As anticipated, the criticality hypothesis states that biological systems benefit from having a critical dynamic regime, not because of the aim of fulfilling a single specific task, but rather, because, in this way, they can better adapt to sudden and/or frequent changes of environment (of tasks). In this sense, criticality is a prerequisite with respect to the task itself. Therefore, we are not interested only in finding the solution with the best possible dynamic regime; rather, we want to know if it is possible to fulfil specific tasks by maintaining a critical dynamic regime. This paper therefore constitutes a step towards a more general study of the combinations of regimes in which the criticality actually offers an evolutionary advantage.

To this end, we make use of a “classic” genetic algorithm, and propose a version which is able to take advantage of the relationship in the RBN that exists among bias, topology, and dynamic regime. As anticipated, the RBNs’ dynamic regime depends on their degree of connectivity and on the bias of the Boolean functions that regulate the activation of each node. In these systems, it is therefore possible to influence the dynamic regime by changing the bias or the topology. This relationship is not necessarily valid for evolved networks; the fact that it has maintained its validity in all our experiments—we checked every GA individual during all GA launches by means of a posteriori test—is an interesting outcome of this study.

We start from systems that already have a critical dynamic regime, and change them gradually (the action of the GA on the Boolean functions). The GA may be run without constraints (and could therefore change the BNs’ bias by influencing the dynamic regime of the system, i.e., “free GA”, or simply “GA”), or it may be constrained to maintain the bias of RBNs present in the first generation. Starting from a generation composed of individuals having similar biases, there are various ways to maintain this situation [52,53]: the GA variant used in this work (called “balanced GA”) randomly chooses the crossover cutoff point among those that lead to the smallest changes of the overall bias of the resulting individuals. Then, if the resulting bias is still different form the desired one, it makes point mutations in order to correct the residual deviations. The positions of these mutations within the string coding the individuals were chosen randomly, whereas their directions (0 → 1 or 1 → 0) were chosen to reach the desired biases. However, we observe that as the GA generations progress, these mutations become less and less frequent). Table 1 shows a general outline of a classic GA and indicates the points where the balanced GA variations are inserted.

### 4.2. The Case Studies

As anticipated, thanks to the use of the balanced version of the GA algorithm, we can show that, starting from critical random BNs (RBNs), populations of (no longer random) BNs can evolve to solve two nontrivial tasks with biological significance by maintaining a critical dynamic regime (Both tasks are not performed by random Boolean networks). At the same time, the comparison between the results of the free GA and those of the balanced GA give rise to interesting observations on the evolutionary process itself.

In particular, we developed systems where:

- the fraction of active genes on attractors is at least a predetermined fraction of the total (“fitness1”)
  ○ we therefore maximize the function $f\left(x\right)=\{\begin{array}{c} \Phi_{1}\;iff\;\Phi_{1}\leq \theta \\ \theta \;iff\;\Phi_{1}>\theta \end{array}\;$. Where not explicitly indicated, *θ* = 0.8.
  ○ the fraction Φ_1_ of active genes is calculated on the states of the attractors found using 10,000 initial conditions
- the fraction of UP genes in an “avalanche” (the fraction of nodes whose activation increases when hit by an avalanche) is at least a predetermined fraction of the total affected nodes (“fitness2”)
  ○ we maximize the function $f\left(x\right)=\{\begin{array}{c} \Psi_{1}\;iff\;\Psi_{1}\leq \delta \\ \delta \;iff\;\Psi_{1}>\delta \end{array}$. Where not explicitly indicated, *δ* = 1.0.
  the fraction Ψ_1_ of UP genes is calculated based on the total of the affected nodes. For an RBN to have a nonzero fitness, it is required that at least 10 avalanches can be carried out in it. In practice, this constraint has always been overcome, and therefore, should not particularly influence the results.

The results shown below refer to experiments whose details are given in Table 2.

## 5. Results

This section may be divided by subheadings. It should provide a concise and precise description of the experimental results, their interpretation, as well as the experimental conclusions that can be drawn.

**Figure 1:**
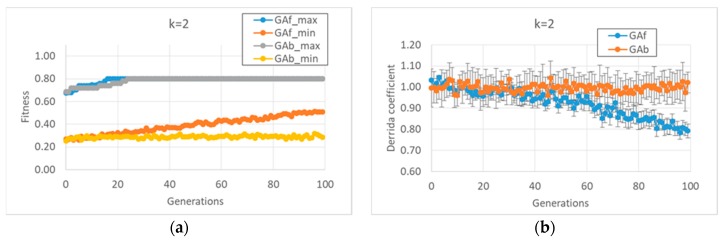
Average or maximum values of some indices, as the 15 evolutionary processes of each GA version-free and balanced, respectively GAf and Gab-progress. (**a**) The maximum of the 15 fitness maxima, and the minimum of the 15 minima, for the two versions of the GA, in cases of populations made of RBNs with *k* = 2 and initial bias = 0.5, which corresponds, on average, to a critical dynamic regime. (**b**) The average Derrida coefficient of free GA and balanced GA (RBNs with *k* = 2); note that the balanced GA succeeds in maintaining the critical dynamic regime. Similar considerations can be made in the case of RBNs with *k* = 3 (data not shown).

In the first case study, it was observed that the “classic” GA strategy (we can define a “strategy” as the kind and sequence of actions performed by the GA on the individuals of the GA population in order to allow the individuals to accomplish the task) changes the dynamic regime of the individuals belonging to the final populations, whereas the balanced GA achieves the same high fitness values by maintaining the starting critical regime, as shown in Figure 1b. The constraint of maintaining a critical regime makes the task more difficult when compared to the “trivial” solutions found by the classic GA; the optimum values of the fitness are reached after the twentieth generation, and low fitness individuals are always present in population. In contrast, the free GA reaches the peak of fitness before the twentieth generation and its populations tend to achieve convergence; see Figure 1a. Similar considerations can be made in the case of *k* = 3 (data not shown).

As anticipated, and indeed remarkably, in these runs (and in all runs of this paper), the relationship between bias and dynamic regime, typical of random BNs, is also maintained by the evolved BNs. In the case of BNs with *k* = 2, it is possible to identify the strategies performed by the two versions of the GA to achieve high fitness.

The free GA acts by increasing the average bias of each BN (see figure MMM and Appendix A). Remarkably, the relationship in random BNs between the bias of Boolean functions and that of “1”s on the attractors is also maintained in this kind of evolved BN. However, this strategy has the consequence of considerably changing the dynamic regime of the final BN; see Figure 1.

**Figure 2:**
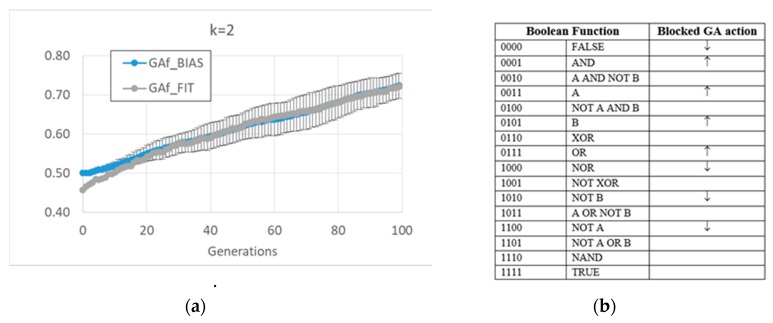
Average or maximum values of some indices, as the 15 evolutionary processes of each GA version-free and balanced, respectively GAf and Gab-progress. (**a**) The maximum of the 15 fitness maxima, and the minimum of the 15 minima, for the two versions of the GA, in case of populations made of RBNs with *k* = 2 and initial bias = 0.5, which corresponds on average to a critical dynamic regime. (**b**) The average Derrida coefficient of free GA and balanced GA (RBNs with *k* = 2); note that the balanced GA succeeds in maintaining the critical dynamic regime. Similar considerations can be made in the case of RBNs with *k* = 3 (data not shown).

The action of the balanced GA is more complex. At a high level, one can observe a change of frequencies in the Boolean functions, aimed at increasing the number of active nodes in time, and maintaining it at a high level once the RBN reaches an attractor rich of “1”s (see Figure 2 and Appendix A for a detailed description). Indeed, a “low-level” interpretation is also possible. Among the functions whose frequency is significantly changed, the frequency of all Boolean functions outputting a “1” increases, and the frequency of all Boolean functions outputting a “0” decreases. The overall RBN behavior therefore leads to an increase, and later to maintaining, a high level of “1”. It should be noted that an analysis in terms of canalizing functions (an interpretative point-of-view common in the RBN literature [57]) is not effective in distinguishing evolved BNs from random BNs (see Appendix A).

Unlike what we saw above, the BNs which are able to reach high fitness values in the second case study (fitness2) show very different final behaviors in case of *k* = 2 or *k* = 3 (In this case, 10 series for each of the two types of GA (and for each of the two input connectivity degree) were made).

**Figure 3:**
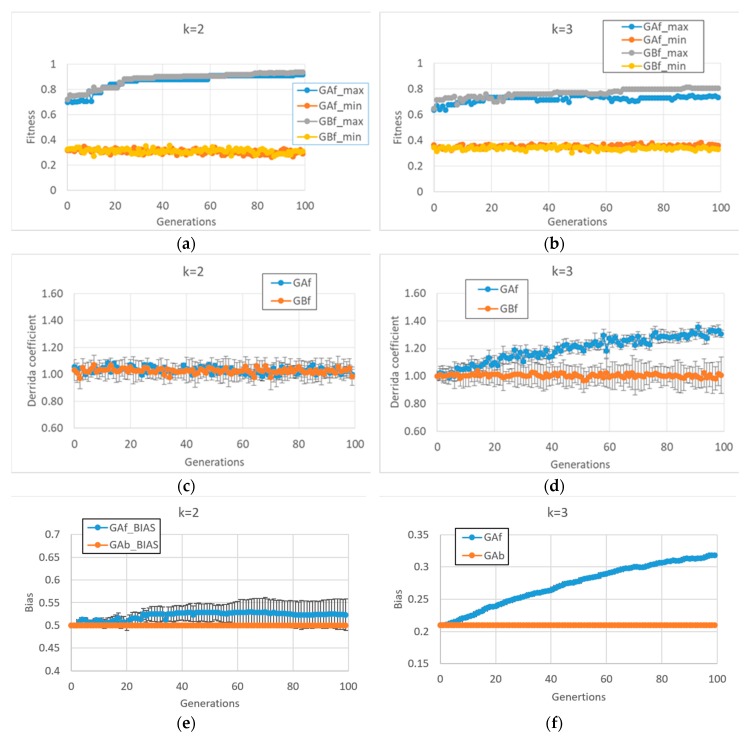
Average or maximum values of some indices, as the 10 evolutionary processes progress. (**a**) The maximum of the 10 fitness maxima, and the minimum of the 10 minima, for the two versions of the GA, in case of populations made of RBNs with *k* = 2 and initial bias = 0.5. (**b**) The same for RBNs with *k* = 3 and initial bias = 0.21. It can be noted that in the case of BNs with *k* = 3, the task is more difficult than with systems with *k* = 2, with the former systems having a higher connectivity, a fact that leads to many more feedback loops which must be be controlled. (**c**) The average Derrida coefficient of free GA and balanced GA (RBNs with *k* = 2). (**d**) The same for RBNs with *k* = 3 and initial bias = 0.21. (**e**) Average population bias of free GA and balanced GA (RBNs with *k* = 2). (**f**) The same for RBNs with *k* = 3 and initial bias = 0.21.

In particular, in cases of BNs with *k* = 2, fitness2 does not seem to require a dynamic regime change per se (see Figure 3c and the corresponding very small bias change in Figure 3e), so the behaviors of the free GA and the balanced GA are very similar Figure 3a. Quite unexpectedly, the same free GA framework applied to BNs with *k* = 3 leads to systems with a disordered dynamic regime (Figure 3d,f). The balanced GA achieves slightly higher fitness by maintaining a critical dynamic regime; in Figure 3d,b, it can be noted that in the case of BNs with *k* = 3, the task is more difficult than with systems with *k* = 2, the former systems having a higher connectivity, a fact that leads to many more feedback loops which must be controlled.

It is interesting to investigate the reasons leading to the different dynamic regime of BNs with *k* = 2 and *k* = 3 in the case of free GA. The reason for this change does not depend on the particular characteristics of the fitness function, but rather, lies in the interaction between the modalities of the “classic” evolutionary process (the free GA) and the types of structures with which it is interacting.

In order to maintain a critical dynamic regime in the case of BNs with *k* = 3, it is necessary to have a different number of “1s” and “0s” in the Boolean functions (a bias of 0.21, or 0.79). The action of the free GA (uniform crossover and uniform mutation) treats zeros and ones equally. Thus, in the case of asymmetry favoring zeros, there are, on average, more mutations that change zeros to ones ones to zeros. This action tends, therefore, to balance the number of ones and zeros; while with *k* = 2, this process is bias-neutral (the symmetry between zeros and ones is already present), with *k* = 3, the process leads far from the parameter region supporting a critical dynamic regime.

In other words, the action of the uniform mutation could (or not) exert a pressure on particular dynamic regimes. It should be noted that this phenomenon occurs independently of (and is superimposed on) the type of function to be performed. By abstracting, the combination of the evolutionary process and the structure to which it is applied can direct evolution per se, in a way that could be only partially connected to the environment that exerts the external evolutionary pressure.

In closing, we can observe that the deviations of the frequency of the Boolean functions with respect to the random case are very small, i.e., almost all below significance thresholds, both in the case of free GA and balanced GA. In future work, we will search for any significant associations between groups of Boolean functions (see Appendix A).

## 6. Conclusions

Let us revisit now the CP that has been introduced and discussed in Section 1, which claims that some dynamical states are advantaged with respect to other states, and that evolution drives living beings towards these “critical” states, which are neither fully ordered nor fully disordered. This statement lends itself, in principle, to experimental verification.

Such testing may, however, be quite difficult, as it would require the ability to analyze the dynamics of the organism with the proper frequency and precision in order to determine whether it is indeed critical. Some subsystems are hardly amenable to such treatment; for example, a direct test for genes might require the availability of data about the activation levels of all the genes, taken at close time intervals on the same organism—something that is still out of reach. However, a suitable alternative is provided by a sophisticated method that combines experimental data with dynamical models; the effects of criticality can be studied in the model, and experimental consequences can be derived. This is the case of gene regulatory networks (GRNs) where, as mentioned in Section 2, it is possible to draw inferences about their dynamical regimes from the available data concerning the distribution of avalanches, or from the study of the dynamics of portions of the network, inferred from available data.

Although the cases analyzed so far do not allow us to draw definitive conclusions, it seems that GRNs in different biological organisms are often found to be critical or close to criticality. As we have seen in Section 3 and Section 4, this is not always the case for artificial networks. This can be attributed to the fact that biological systems live in a changing environment, while artificial systems are often confronted with constant tasks. We will undertake a thorough investigation of the conditions which might lead artificial systems towards criticality in the future.

The approach we have taken in this paper is that of assuming that criticality can be a long-term advantage, as suggested by the analysis of biological networks, and of imposing it as a constraint. So, we have shown that constraining a Boolean gene regulatory network to remain critical does not impair its ability to solve two nontrivial tasks which have sound biological motivations. Therefore, it is plausible that critical systems can adapt to deal with different environments while maintaining their criticality, although, of course, the performance of the two tasks does not prove that this is always the case.

Beside these considerations, it is important to highlight the fact that the same action of evolutionary processes (crossover and mutation) on individuals with different structures (for example, BNs with *k* = 2 or *k* = 3) could change (or not) their dynamical regimes, irrespective of the external environment (i.e., of the task to be accomplished).

An important aspect that is worth mentioning is that the modified GA actually acts on the values of the bias *p,* while the number of incoming links per node is kept constant. There are a couple of values of k and p which are usually associated with critical behaviors (e.g., *k* = 2, *p* = 1/2, or *k* = 3, *p* = 0.21), but these associations are valid *strictu sensu* only for random BNs, while the networks which we find at the end of the artificial evolution are no longer purely random, since they have been selected for their performance. However, in all the evolved networks, we measured the Derrida parameter and checked that it was indeed equal or close to one for those couples of parameter values that would guarantee criticality in RBNs. Therefore, we can claim that the evolved networks are truly dynamically critical, according to the classical definition which associates this property with the fact that the Derrida parameter equals 1. The fact that artificial evolution maintains true dynamical criticality, while the GA only acts on the fraction of 0 s and 1 s, is worthy of further exploration.

we should mention that Boolean networks are nonergodic systems, and different notions of criticality can be proposed, as discussed in [29]. The classical Derrida parameter is measured by computing the distance between initially close states, which are chosen at random, without any constraints. In contrast, it is possible, e.g., to consider only states which belong to an attractor, and to measure the average, taken only on those states and their neighbors, of the Derrida parameter. Study of the effects of different restrictions to the possible initial states is outside the scope of the present paper, but will be addressed in future work.

## Figures and Tables

**Table 1:** The general scheme of the used GAs (#G denotes the number of elements in set G). Steps 6 and 7 highlight the differences between free and balanced GA [52].

Step	Step Description	
1	create network topology (it will be the same for all the networks for all generations)	
2	create the first population G of networks (Boolean functions are generated at random with bias *p*)	
3	for each network in G, compute the fitness	
4	select the set E of the individuals with the highest fitness, which will be passed unaltered to the next generation (elitism)	
5	select parents for the individuals of the new generation, with probabilities proportional to their fitness (their number being equal to #G−#E)	
6	generate the set G’ by applying single-point crossover to the selected parents in G with a given probability (otherwise parents pass unmodified in the new population)	BALANCED GA. generate the set G’ by applying single-point crossover to the selected parents in G with a given probability (otherwise, parents pass unmodified to the new population). The crossover cutoff point is chosen among those that least change the overall bias of the resulting individuals
7	generate the set G” by applying single-point mutation to individuals in G’ with a fixed (small) probability	BALANCED GA. generate the set G” by applying single-point mutation to individuals in G’ with a fixed (small) probability. Add some mutations in order to maintain the children’s bias close to the parents’ bias
8	generate the new population of networks G = E∪G”	
9	if the termination condition has not been met, return to step 3	
10	End	

**Table 2:** The parameter values used during simulations. ** In order to characterize the systems under analysis, when necessary (searching for attractors, determination of the Derrida parameter), we used 10,000 initial conditions.

System	Parameter	Value
GA	number of generations	100
GA	number of RBNs in population	100
GA	crossover probability	0.7
GA	mutation probability per node	0.02
GA	number of individuals in elite E	3
RBN	number of nodes per RBN	50
RBN	number of inputs per node *	2 (3)
RBN	average initial bias in initial population ^1^	0.5 (0.79) (0.21)
RBN	number of initial conditions per RBN ^2^	10,000

^1^ The RBNs can have 2 or 3 input links for each node. In the case of 2 links, we always used bias = 0.5, while with 3 connections, we used bias = 0.21 or bias = 0.79 (both bias in RBNs ensure critical dynamic regimes).^2^ In order to characterize the systems under analysis, when necessary (searching for attractors, determination of the Derrida parameter), we used 10,000 initial conditions.

**Figure A1:**
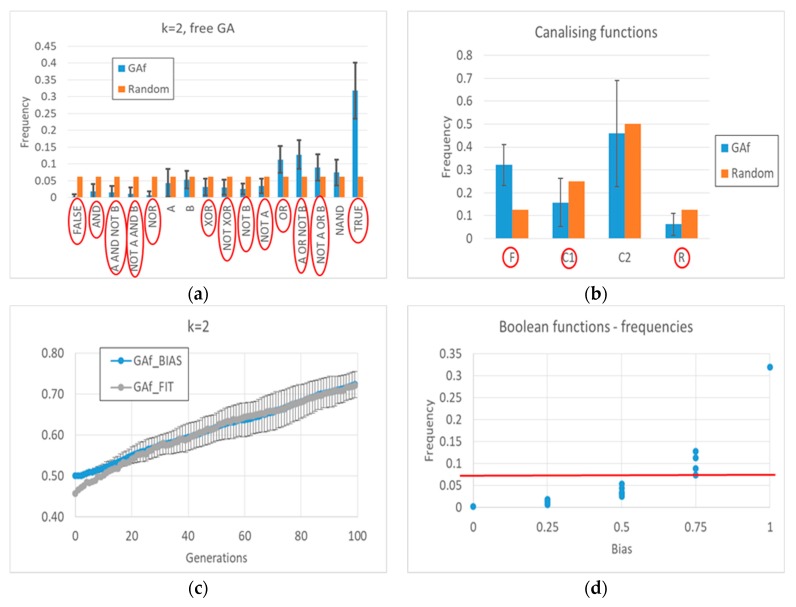
(**a**) Probability occurrence for each Boolean function, in the case of random and evolved RBN-the bars for each measure indicate the standard deviation of the distribution). Boolean functions are ordered by increasing bias. (**b**) The same of (**a**), by using the four canalization classes; F fixed functions; C1 canalizing function in one input channel and C2 in two input channels; R, the XOR and NOT XOR functions. (**c**) Average population bias, and average fitness. In random BN the number of active genes is on average equal to the bias of the Boolean functions: this property seems maintained by the BN evolved through the free GA. (**d**) Frequency for each Boolean function in the case of random (red line) and evolved RBN: the Boolean functions are categorized by using their bias. In (**a**) and (**b**) parts of the figure the variables deviating from independence at level of 0.02 (stronger evidence) are highlighted by red circles, and those deviating at 0.05 level (weaker evidence) by blue circles.

**Figure A2:**
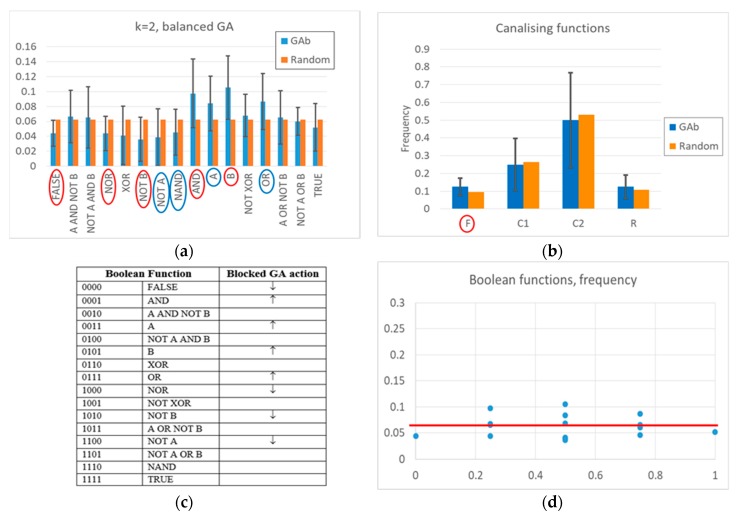
(**a**) Frequency of each Boolean function, in the case of random and evolved RBN-the bars for each measure indicate the standard deviation of the distribution. Boolean functions are ordered based on the presence of a zero or a one in correspondence of the input consisting of a double “one”. (**b**) The same of (**a**), by using the four canalization classes; F fixed functions; C1 canalizing function in one input channel and C2 in two input channels; R, the XOR and NOT XOR functions. (**c**) A table illustrating the strategy implemented by the balanced GA-the table shows only the trend of growth or decrease of the Boolean functions whose frequency has significantly changed. (**d**) Probability occurrence for each Boolean function in the case of random (red line) and evolved RBN: the Boolean functions are categorized by using their bias. In (**a**) and (**b**) parts of the figure the variables deviating from independence at level of 0.02 (stronger evidence) are highlighted by red circles, and those deviating at 0.05 level (weaker evidence) by blue circles.

## Appendix A

In this appendix we discuss whether it is possible to extract useful information about the action of the two GA variants (free GA and balanced GA), by analyzing the best individuals of each GA series.

In order to reduce the inevitable specificity of those individuals-and in search of general rules-we generated a generic profile for each series of GA. For example, we have put together all the Boolean functions of the winning individuals of the 15 launches of the free GA (*k* = 2, fitness1 (We remember that fitness1 concerns the search for BNs able to have a certain fraction of ones in the attractors, while fitness2 concerns the search for BNs able to have a certain fraction of UP genes in the avalanches (see the main text for more details)), assuming that these 15 individuals were all instances of the same resolutive logic (indeed, they evolved to solve the same problem). From this, we have been able to infer an average and a standard deviation for the frequency of each Boolean function, typical of the solutions of the problem. A t-test [58] can be applied to these data, to investigate the null hypothesis that the population mean is equal to a specified value (in our cases, the typical Boolean functions frequency of the initial random BNs).

After that, we repeated the same operation for the balanced GA (*k* = 2, fitness1), for the free GA (*k* = 3, fitness1), for the balanced GA (k = 3, fitness1). The same in the case of fittness2.

In the case of fitness2 there are very few deviations from the null hypothesis (identity with random BNs), with very low confidence levels; nothing is observable by using the Boolean functions canalizing classes. Moreover, no obvious strategies (We remember that we defined a “strategy” as the kind and sequence of actions performed by the GA on the individuals of the GA population in order to allow the individuals to accomplish the task) are evident: in this case it is perhaps necessary to go beyond the mere distribution of Boolean functions and check the associations between them, an analysis that we leave to further works.

In the case of fitness1, on the other hand, it is possible to highlight numerous deviations, and to infer solution strategies based on these observations. In this appendix we present the results for the case of BNs with *k* = 2.

### A.1. Free GA

We made 15 series for each of the two types of GA, starting from initial populations composed of individuals with an average bias equal or near to 0.5. The initial individuals therefore exhibit critical dynamic regimes.

At the end of the evolutionary process the frequencies of the Boolean functions belonging to the best individuals are different from the homogeneous case (all Boolean functions having the same frequency): we therefore make use of the t-test [58], to investigate the null hypothesis that each frequency mean is equal to the corresponding value in homogeneous case. We make use of the two-tailed test: in all the following figures the variables deviating from independence at confidence level of 0.02 (stronger evidence) are highlighted by red circles, and those deviating at 0.05 confidence level (weaker evidence) by blue circles.

Figure A1 shows the evidences supporting the hypothesis of a systematic shift of the bias of the Boolean functions, in the case of free GA (the Boolean functions are ordered by increasing bias). A shift is also visible at the level of the canalizing functions Figure A1b.

### A.2. Balanced GA

Figure A2 shows the evidences supporting the hypothesis of a systematic shift of the frequency of the Boolean functions, in the case of balanced GA. The shift is almost not visible at the level of the canalizing functions Figure A2b.

The emerging strategy in the case of the balanced GA is slightly more sophisticated than the strategy emerging in the case of the free GA, and involves the change of frequency of some Boolean functions, aimed at increasing in time the number of active genes-and maintaining it once the RBN is on an attractor. By keeping a high level description:

- the frequency of FALSE function is decreased
  ○ this function tends to introduce zeros-and is therefore to be removed
- the frequency of AND function is increased, and that of its opposite NAND is decreased
  ○ when both parent nodes are turned on, the downstream node remains turned on, and the opposite has not to happen
- the frequency of COPY A and COPY B is increased, whereas that of their opposite NOT A and NOT B is decreased
  ○ at steady state in these systems there are many activated nodes: in such situation the downstream nodes copy this high activity-the opposite has not to happen
- the frequency of OR is increased, and that of its opposite NOR is decreased
  ○ it is sufficient that one of the parent nodes is active to activate-the opposite has not to happen

Indeed, a “low-level” interpretation of this strategy is possible: among the functions whose frequency is reported as significantly changed, the frequency of all Boolean functions having a “1” in correspondence of the input consisting of a double one has increased, and the frequency of all Boolean functions having a “0” in correspondence of the input consisting of a double one has decreased. The overall RBN behavior therefore leads to increase and then to maintain a high level of “1”, while maintaining a critical dynamic regime.

Note that this action is almost not visible at the level of canalizing functions, and that the classification based on bias is indeed very confusing (indeed, the average bias is not changed—Figure A2).
